# Validation of the Powder Metallurgical Processing of Duplex Stainless Steels through Hot Isostatic Pressing with Integrated Heat Treatment

**DOI:** 10.3390/ma15186224

**Published:** 2022-09-07

**Authors:** Louis Becker, Jonathan Lentz, Berenice Kramer, Anna Rottstegge, Christoph Broeckmann, Werner Theisen, Sebastian Weber

**Affiliations:** 1 Materials Technology, Ruhr-University Bochum, 44801 Bochum, Germany; 2 Institute of Applied Powder Metallurgy and Ceramics, RWTH Aachen e. V., 52062 Aachen, Germany

**Keywords:** duplex stainless steels, CALPHAD, phase equilibria, hot isostatic pressing, integrated heat treatment

## Abstract

Duplex stainless steels exhibit an excellent combination of corrosion resistance and strength and are increasingly being manufactured through powder metallurgy (PM) to produce large, near-net-shaped components, such as those used for offshore applications. Hot isostatic pressing (HIP) is often used for PM production, in which pre-alloyed powders are compacted under high pressures and temperatures. Recent developments in HIP technology enable fast cooling as part of the process cycle, reaching cooling rates comparable to oil quenching or even faster. This enables the integrated solution annealing of duplex stainless steels directly after compaction. In contrast to the conventional HIP route, which requires another separate solution annealing step after compaction, the integrated heat treatment within the HIP process saves both energy and time. Due to this potential gain, HIP compaction at a high pressure of 170 MPa and 1150 °C with integrated solution annealing for the production of duplex stainless steels was investigated in this work. Firstly, the focus was to investigate the influence of pressure on the phase stability during the integrated solution annealing of the steel X2CrNiMoN22-5-3. Secondly, the steel X2CrNiMoCuWN25-7-4, which is highly susceptible to sigma phase embrittlement, was used to investigate whether the cooling rates used in the HIP are sufficient for preventing the formation of this brittle microstructural constituent. This work shows that the high pressure used during the solution heat treatment stabilizes the austenite. In addition, it was verified that the cooling rates during quenching stage in HIP are sufficient for preventing the formation of the sigma phase in the X2CrNiMoCuWN25-7-4 duplex stainless steel.

## 1. Introduction

Duplex stainless steels (DSS) are characterized by an excellent combination of strength and toughness with a high corrosion resistance [1]. There are numerous applications for which this combination of properties is required, and DSS are therefore found in maritime applications, including seawater applications. These include, for example, ship propellers or pipe nodes and distributors in offshore applications on the seabed [2]. Here, a resistance to pitting corrosion is especially required; thus, steels with a pitting resistance equivalent number of >32 are used [3]. In applications, weaknesses usually occur in the welded joints of complex-shaped components (such as the above-mentioned pipe nodes used for offshore applications). This is because the desired precipitation-free microstructure of austenite and ferrite cannot always be precisely adjusted in this case. For example, the brittle intermetallic precipitation of the Mo- and Cr-rich sigma phase can occur [4]. The existence of this phase leads to a local decrease in the corrosion resistance, strength, and toughness in the heat-affected zone of the welds [5].

This can be remedied by an innovative, powder-metallurgical manufacturing process, in which complexly shaped raw joints of large dimensions and different wall thicknesses that are close to final contour can be produced [6]. Sheet metal capsules already formed close to the final contour are filled with DSS metal powder and compacted into solid components by hot isostatic pressing (HIP). In a subsequent processing step, these components are solution annealed to obtain a duplex microstructure consisting of 50% austenite and 50% ferrite and rapidly quenched to suppress nucleation- and diffusion-dependent precipitation of the sigma phase at temperatures below 900–800 °C. Barros et al. [7] investigated this process using the duplex stainless steel X2CrNiMoN22-5-3 and compared its microstructure and properties with those of steel in a previously hot-rolled material condition. A finer and more uniform austenite distribution was detected in the PM-produced duplex steel. In addition, the microstructure, as well as the mechanical properties, were isotropic [7]. Recent advancements in HIP plants enable the integration of fast cooling with HIP [8]. This function is also called uniform-rapid quenching (URQ) and is based on internal gas cooling by convection. A heat sink for cooling the gas is installed inside the pressure vessel so that the gas remains in the vessel, leading to a significant acceleration of the cooling rate. Supposedly, cooling rates of up to 3000 K/min are attainable [9,10]. This quenching technique can be used in an integrated HIP treatment, where the steps of the HIP compaction and solution annealing are combined in only one process. Compared to the process of “HIP + subsequent solution annealing”, the integration of solution annealing into the HIP process can lead to the following advantages: (a) a shortened process time by up to 80%, (b) the saving of energy by eliminating the need for reheating, (c) lower thermal stresses due to gas cooling, which favors distortion- and crack-free quenching, and (d) prevention of grain growth, which leads to better mechanical properties [11].

In the work of Deng et al. [11] and Chen et al. [12], the process technology of integrated heat treatment with HIP has already been addressed with respect to two different material groups. Deng et al. [11] focused on the duplex stainless steel 318LN and were able to simulate its densification and microstructure formation during HIP. With regard to the microstructure formation, they referred in particular to the brittle sigma phase. With the simulation approach developed in this work, the sigma phase formation during HIP and after different cooling rates, and thus also that of the integrated heat treatment, were predicted and validated using experimental methods [11]. Chen et al. [12] investigated the integrated heat treatment with HIP using a lamellar and a duplex structured TiAl alloy and found that the materials that were heat-treated under high pressures had better mechanical properties compared to those subjected to the conventional manufacturing route (HIP with subsequent heat treatment at an atmospheric pressure) [12].

Another interesting area of application of solution annealing integrated with HIP is the thermal post-treatment of additively manufactured duplex stainless steels. In several works, duplex stainless steels, such as the X2CrNiMoN22-5-3 steel and the X2CrNiMoN25-7-4 steel, have already been additively manufactured using laser powder bed fusion (PBF-LB/M) [13,14,15,16,17,18]. Due to the high solidification and cooling rates characteristic of this process, the components are in a predominantly ferritic phase state, which means that subsequent heat treatment is required to adjust the phase mixture of the 50 vol.% austenite and 50 vol.% ferrite. In addition, the formation of various defects, such as gas pores or lack-of-fusion pores, is possible, which negatively affect the properties of the components and should therefore be kept as low as possible. To decrease the defect density, the components are often subjected to HIP post-treatment. Mirz et al. [17] subjected the PBF-LB/M-processed X2CrNiMoN22-5-3 duplex stainless steel to HIP post-processing after fabrication, which significantly reduced the porosity [17]. However, the steel had to be solution annealed afterwards, which would not have been necessary if solution annealing integrated with HIP had been conducted. 

The analysis presented makes it clear that the solution annealing of steel integrated with HIP can offer additional value compared to the conventionally produced grades, but also with regard to the post-processing of additively manufactured steels. Against this background, two general questions regarding the integrated solution annealing arise, which are investigated in the following pages:

1. How does the pressure at work during the HIP process affect the phase stability and, thus, the integrated solution annealing of duplex stainless steels? This question is to be investigated using X2CrNiMoN22-5-3, first by compacting it powder metallurgically by means of HIP, and then by solution annealing it directly in HIP combined with URQ. A subsequent conventional solution-annealed X2CrNiMoN22-5-3 steel serves as a reference.
2. It remains to be investigated whether the rapid cooling in HIP using the modern processes of URQ is sufficient for preventing the formation of the sigma phase after solution annealing and thus avoiding the embrittlement of the workpieces. For this purpose, the steel X2CrNiMoCuWN25-7-4 was analyzed, which shows a higher susceptibility to sigma phase precipitation compared to the steel X2CrNiMoN22-5-3 (1.4462), due to its higher Cr and Mo contents.

## 2. Materials and Methods

### 2.1. Sample Production

With the aim of investigating the influence of high pressure on austenite stability, investigations were carried out using the duplex stainless steel X2CrNiMoN22-5-3. Furthermore, this work investigated whether it is possible to suppress the formation of the sigma phase in URQ cooling following the HIP process. The duplex steel X2CrNiMoCuWN25-7-4 was chosen for these investigations because it has a high tendency towards sigma phase formation during cooling.

For both investigations, powder was used as the starting material, the chemical composition of which can be viewed in Table 1. The X2CrNiMoN22-5-3 and X2CrNiMoCuWN25-7-4 steel powders, which were purchased from Carpenter, had a fraction ranging from 0.1 to 500 µm.

These pre-alloyed metal powders were welded gas-tight in cylindrical capsules (for X2CrNiMoN22-5-3: 44.5 mm in both diameter and height; for X2CrNiMoCuWN25-7-4: diameter 70 mm and height 90 mm), evacuated, and compacted in the HIP (Quintus QIH9) and subjected to integrated solution annealing. The process parameters used in the framework of the HIP and subsequent solution annealing are given in Table 2. To enable complete densification, a temperature of 1150 °C (T_hold,1_) was first maintained for 180 minutes (t_hold,1_) at a pressure of 170 MPa (p_hold,1_). Subsequently, inter-critical solution annealing was carried out at 1075 °C (T_hold,2_) for the X2CrNiMoN22-5-3 steel and at 1050 °C (T_hold,2_) for the X2CrNiMoCuWN25-7-4 steel, respectively, for 60 minutes (t_hold,2_) at a pressure of 170 MPa (p_hold,2_). In all cases, subsequent URQ quenching under a pressure of 100 MPa took place. The materials used in this application are listed in Table 2, with the abbreviation HIP_ISA, which stands for HIP with integrated solution annealing. In order to enable the mapping of the pressure’ influence on the austenite stability during the solution annealing of the steel X2CrNiMoN22-5-3, it was necessary to subsequently produce a reference condition of this steel according to the conventional heat treatment procedures. For this purpose, a further heat treatment step was carried out after the aforementioned densification and solution annealing with HIP (post-HIP SA). This was carried out in an inert gas furnace in an Ar atmosphere at atmospheric pressure (p_hold,3_), at a temperature of 1075 °C (T_hold,3_) for 60 minutes (t_hold,3_). The quenching was carried out in water. This processing state is entitled HIP+CSA in Table 2, which stands for HIP and conventionally solution-annealed.

**Figure 1:**
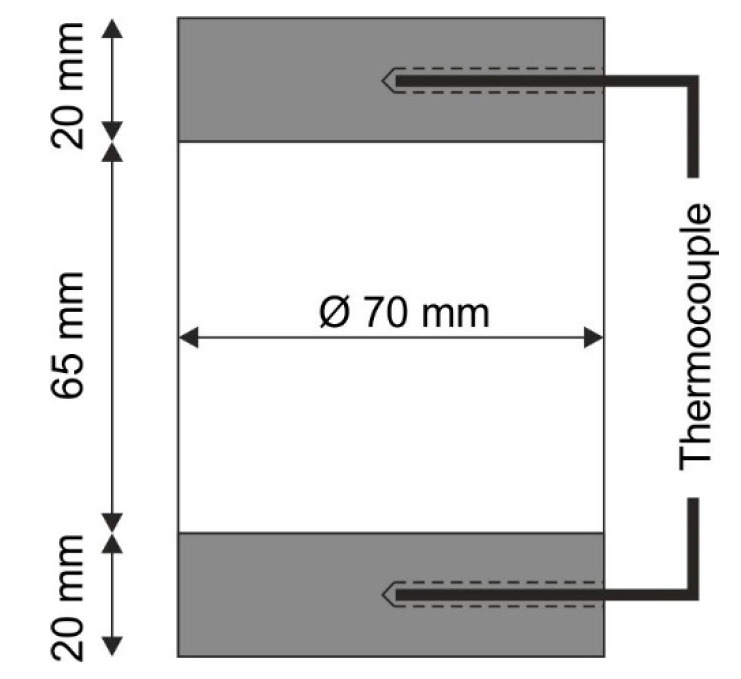
Geometry of the HIP capsule used for the in situ temperature measurements of the steel X2CrNiMoCuWN25-7-4.

Since the actual cooling rate is important in the context of the investigations of the sigma phase formation during quenching in HIP, a special capsule geometry was used for the densification of the steel X2CrNiMoCuWN25-7-4. In order to facilitate the in situ measurement of the capsule temperature and, thus, the quenching effect achieved by means of the URQ technique in the HIP capsules, steel capsules (X6CrNiMoTi17-12-2) with a thicker design of the bottom plates (Figure 1) were used. In this way, thermocouples (type B, platinum30-rhodium/platinum6-rhodium) could be inserted via holes in the bases and lids of the HIP capsules. From the hot isostatically pressed powder, 10 notched impact specimens (notched Iso-V geometry 55 × 10 × 10 mm^3^) were taken from the capsule in each longitudinal direction by wire-eroding.

### 2.2. Experimental Investigation of the Austenite and Ferrite Contents

The characterization of the microstructure was carried out using metallographically prepared cross-sections. The preparation method included cutting, grinding (up to 1000 mesh), and polishing with a diamond suspension of up to 1 µm.

For the quantitative measurements of the austenite and ferrite contents, as well as microstructural investigation, the electron backscatter diffraction technique (EBSD) was used. For EBSD studies, a further polishing step was carried out using an oxide particle suspension with a mean particle diameter of 0.25 µm. The EBSD investigation was performed using a MIRA3 scanning electron microscope (company: Tescan, Brno, Czechia) equipped with a Nordlys nano detector and Aztec software (company: Oxford, Oxford, UK). The acceleration voltage of the electron beam was 20 keV, and the working distance was 17 mm. The images were recorded at a step distance of 0.3 μm, a binning factor of 4 × 4, and an exposure time of 2 min. In order to detect the differences in the chemical composition between the austenitic and ferritic phases, energy-dispersive X-Ray spectrometry (EDS) (company, Oxford, Oxford, UK) measurements were also carried out over the course of the EBSD investigations. An Aztec Energy Advanced System from Oxford Instruments was used for these measurements.

In addition, two further techniques were used to quantify the austenite/ferrite contents, which feature a higher interaction volume according to the literature [18]. A magneto-inductive quantification was performed using Feritscope^®^ of type FMP 30 from the company Fischer (Mödling, Austria). Three measurements were performed for each material, and the mean value was determined. Based on the EBSD measurements, it could be assumed that the microstructure consisted only of austenite and ferrite. For this reason, the austenite content was determined from the Feritscope^®^ measurements by calculating the 100 vol.% - measured vol.% of ferrite.

Furthermore, a quantitative phase analysis was performed by the X-Ray diffraction technique using the diffractometer model µ-X360n (company Pulstec, Hamamatsu, Japan), which emits CrKβ radiation (λ = 0.22898 nm). In this technique, the X-ray beam is emitted with a tube voltage of 30 kV and focused by a 2 mm collimator. The distance between the radiation exit window and the sample surface (working distance) was 20 mm ± 0.5 mm. The X-ray incidence angle was 18°. This device works according to the Debye–Scherrer method, which means that the reflecting X-rays are detected by a 2D detector. In the setting described, only the Debye–Scherer rings of the (220) γ-reflection (2 ϴ = 128.8°) and the (221) α-reflection (2 ϴ = 156.4°) were detected, which served as the basis for the phase quantification. This was carried out according to ASTM E975-13 [19].

### 2.3. Charpy Impact Testing

In addition to the microstructural investigation and phase analysis, the presence of the sigma phase in the steel X2CrNiMoCuWN25-7-4 was investigated by impact testing. Impact testing is designed to reveal a decrease in toughness in the presence of the sigma phase p (even at levels below the resolution limit of the methods described in Section 2.2). Charpy impact tests were carried out according to DIN EN ISO 148 on specimens with a V-notch (notch depth 2 mm). The specimens had a square cross-section of 10 mm and a length of 55 mm. The tests were carried out using a Wolpert pendulum impact tester at a maximum impact energy of 300 J. The notched impact hammer had an impact velocity of 5.5 m/s. The notched impact effect performed was determined by a drag indicator. The friction effect of 1 J caused by the drag indicator was included in the evaluation. The test temperatures were −46 °C, −20 °C, and room temperature (RT). Samples at temperatures below room temperature were set by a mixture of liquid nitrogen and ethanol and monitored with a thermometer. The cooled samples were tested immediately after removal from the cooling mixture. A total of 4 low temperature samples were tested, and 3 room temperature samples were tested. SEM images of the fracture surfaces were then taken using the scanning electron microscope mentioned in Section 2.2.

### 2.4. Thermodynamic Calculations

For the thermodynamic calculation of the pressure influence on the phase composition, the software ThermoCalc (version 2022a) (Thermo-Calc, Solna, Sweden) and the database TCFe10 were used. For the calculations, the phases of ferrite, austenite, liquid, sigma, and Chi, as well as a substance quantity of 1 mol and the chemical compositions given in Table 1, were used. The influences of the pressure and temperature on the phase stability were calculated accordingly.

## 3. Results and Discussion

### 3.1. Thermodynamic Influence of the HIP Pressure on the Austenite Stability

**Figure 2:**
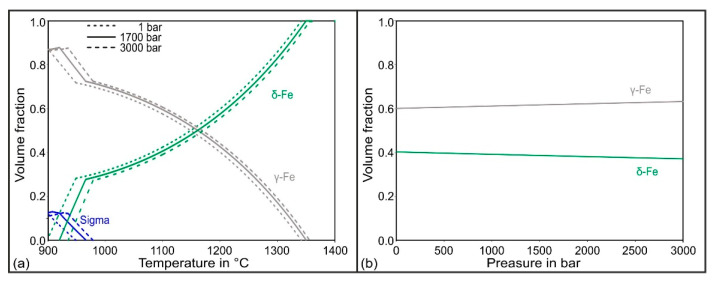
Thermodynamic calculations of the pressure influence on the phase stability during the solution annealing of the steel X2CrNiMoN22-5-3: (**a**) phases over the temperature for 1 bar, 1700 bar, and 3000 bar; (**b**) phases over the pressure.

Before discussing the experimental results regarding the influence of pressure on the phase stability, the thermodynamic influence of the HIP pressure on the austenite stability is examined in this section based on the thermodynamic calculations. These were carried out for the duplex stainless steel X2CrNiMoN22-5-3. The chemical composition used for the calculations corresponds to that presented in Table 1. Firstly, as seen in Figure 2a, three different phase quantity diagrams were superimposed, corresponding to the pressures of 1 bar, 1700 bar, and 3000 bar. In the considered temperature range of 900 °C to 1400 °C, the sigma, austenitic, and ferritic phases were stable. At the solution heat treatment temperature of 1075 °C used in this work, on the other hand, only the ferrite and austenite were thermodynamically stable. Figure 2b shows the volume fractions of the ferritic and austenitic phases as a function of the pressure at the solution heat treatment temperature of 1075 °C. At the atmospheric pressure of 1 bar, 59 vol.% austenite was stable. This proportion increased slightly with increasing pressure, so that, at 1700 bar, 62 vol.% austenite was stable. The thermodynamically stable austenite content continued to increase slightly up to a pressure of 3000 bar, where it reached a value of 63 vol.%.

### 3.2. Experimental Studies on the Influence of Pressure on the Microstructural Austenite/Ferrite Stability

**Figure 3:**
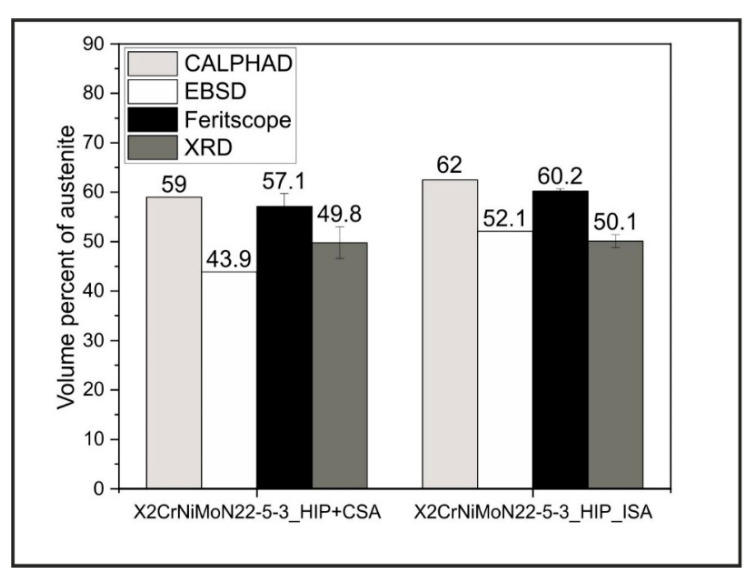
Overview of the austenite contents determined by CALPHAD, EBSD, Feritscope^®^, and XRD for the two considered conditions: X2CrNiMoN22-5-3_HIP+CSA (solution annealing at 0.1 MPa and 1075 °C) and X2CrNiMoN22-5-3_HIP+ISA (solution annealing at 170 MPa and 1075 °C).

The thermodynamic calculations shown in Section 3.1 suggest an influence of pressure on the phase stability during the solution annealing of the duplex stainless steel X2CrNiMoN22-5-3. In this work presented in this section, we verified this suggestion by means of experimental investigations using different phase quantification methods applied to the two different solution-annealed X2CrNiMoN22-5-3 steels. Figure 3 shows the austenite contents determined using EBSD, Feritscope^®^, and XRD. In addition, the austenite contents calculated using CALPHAD are shown, so that we may draw a comparison. First, the results are presented, and then they are compared.

**Figure 4:**
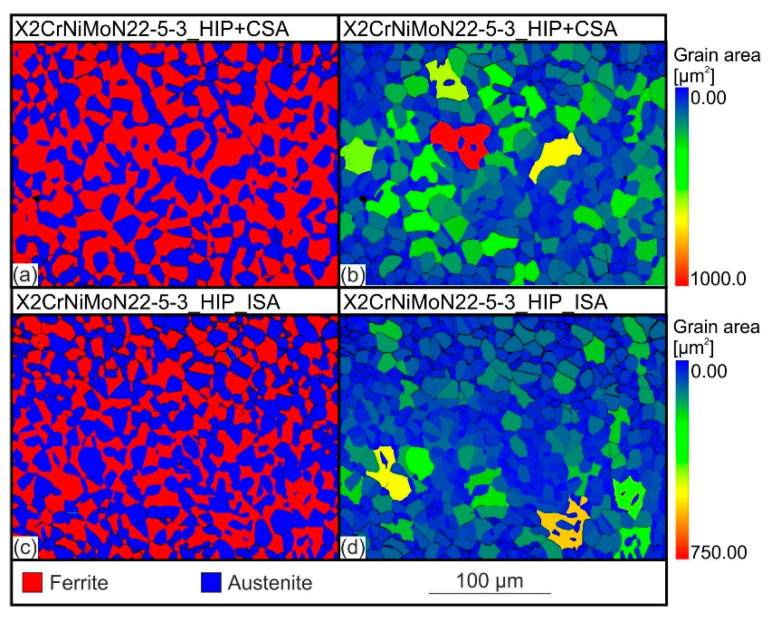
EBSD phase images (**a**,**c**) and grain size mappings (**b**,**d**) of the X2CrNiMoN22-5-3_HIP+CSA and X2CrNiMoN22-5-3_HIP_ISA steels.

All the austenite contents shown in Figure 3, regardless of the quantification method used, show that the sample X2CrNiMoN22-5-3_HIP_ISA had a higher austenitic phase content compared to the sample which was subsequently solution-annealed at an atmospheric pressure, X2CrNiMoN22-5-3_HIP+CSA. Both the austenite levels and the magnitude of the differences between the two material conditions changed depending on the method used. The austenite contents determined by EBSD, based on the phase images in Figure 4a,c, were the lowest among the considered methods, with an austenite content of 43.9 vol.% forX2CrNiMoN22-5-3_HIP+CSA and a content of 52.1 vol.% for X2CrNiMoN22-5-3_HIP_ISA. In contrast, the austenite contents which were determined by detecting the ferrite content with the aid of the Feritscope^®^ showed the highest values (X2CrNiMoN22-5-3_HIP+CSA: 57.1 vol.% and X2CrNiMoN22-5-3_HIP_ISA: 60.2 vol.%). The austenitic phase fractions measured by XRD were between the austenite levels obtained from EBSD and Feritscope^®^. The XRD measurements showed only a small difference in the austenite contents, at 0.3 vol.%, between X2CrNiMoN22-5-3_HIP_ISA and X2CrNiMoN22-5-3_HIP+CSA. The fact that the austenite contents varied depending on the characterization method used can be attributed to method-related differences in the quantification of the phase fractions, as well as differences in the size of the volume or surface area investigated, respectively [18]. In EBSD investigations, only a sample area of 62500 µm2 can be measured, while the area investigated in the XRD analysis was 50 times larger. With the Feritscope^®^, on the other hand, the measurement is not only surface-sensitive but also volumetric. In this case, the considered sample area corresponded to a volume of 10 mm3. As this was the largest sample range considered among all the phase quantification methods used in this work, the results of the Feritsope^®^ were considered most reliable for the phase quantification. Compared to the austenite contents calculated using the CALPHAD method, the contents determined using the Feritscope^®^ were approx. 2 vol.% lower. However, this deviation still corresponds to a strong agreement between the thermodynamic calculations and the real existing phase contents. For this reason, the measurement results confirm the influence of pressure on the austenitic and ferritic phase fractions, as suggested by the calculations in Section 3.1.

The influence of pressure on the phase stability of Fe-based materials has been the subject of only a small number of publications. In principle, the slope of the equilibrium line of two phases, such as ferrite and austenite, can be described by the Clausius-Clapeyron equation (see Equation (1)) [20].

(1) $$\left(\frac{\mathrm{dp}}{\mathrm{dT}}\right)=\frac{\Delta H}{\Delta V\;T}$$

Here, ΔH is the enthalpy change with a phase change, ΔV is the volume change with a phase change, and p and T stand for the pressure and temperature on the equilibrium line, respectively. This equation clearly shows that phase transformations take place with increasing pressure in the direction of a lower specific volume. Compared to the body-centered cubic (bcc) ferrite lattice, the face-centered cubic (fcc) austenitic lattice is more densely packed and has a lower specific volume, which is why, according to Equation (1), austenite is stabilized with increasing pressure. Hilliard [21] demonstrated this through an FeC phase diagram plotted against the temperature and pressure. There, a shift of the ferrite-to-austenite conversion line towards lower temperatures with increasing pressure could be seen. The effect of this phenomenon has, thus far, been observed mainly in martensitic hardening, where the stabilization of the austenite occurs as a result of the influence of pressure. In martensitic hardenable steels, the stabilization of austenite by the high pressure applied in the HIP process has already been studied and, here, was used to improve the hardenability [22]. As a result, higher martensite and/or retained austenite contents were detected after hardening at increasing pressures. In addition to this phenomenon, it was shown on the basis of the results collected here that a phase shift in favor of the austenite also occurred during the solution annealing of the duplex stainless steels due to the increased pressure.

**Figure 5:**
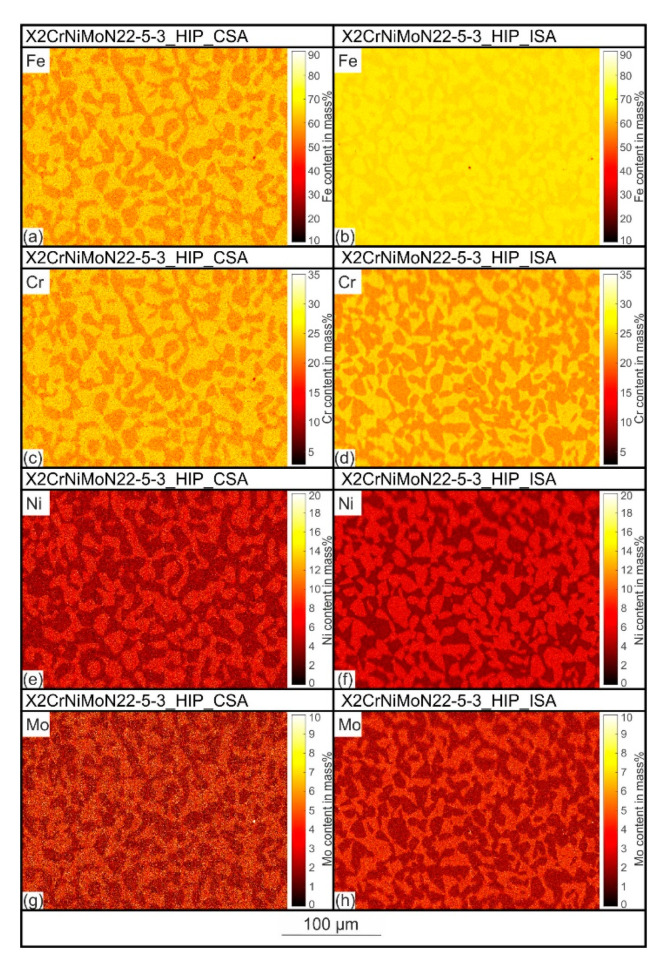
Element distributions of the X2CrNiMoN22-5-3_HIP+CSA (**a**,**c**,**e**,**g**) and X2CrNiMoN22-5-3_HIP_ISA steels (**b**,**d**,**f**,**h**) determined by EDS.

In addition to the phase quantification, the EBSD mappings shown in Figure 4a,c, provide important information about the microstructure and differences between the two steel states considered. In both cases, a typical two-phase duplex microstructure were detected. The austenitic and ferritic phase fractions were evenly distributed over the cross-sections of the samples. Figure 5 shows the element distributions associated with the EBSD images in Figure 4. In the microstructure of X2CrNiMoN22-5-3_HIP+CSA as well as X2CrNiMoN22-5-3_HIP_ISA, a partitioning of the elements Fe, Cr, Ni, and Mo was detected. The ferrite-stabilizing elements Cr and Mo are predominantly found in ferrite, while the austenite-stabilizing element Ni is more prevalent in austenite. The differences in the chemical composition between ferrite and austenite are based on the different solid solubility potentials of the alloying elements in the bcc and fcc lattices [23] and have been observed in numerous studies (e.g., in [24]). There was no significant difference in the degree of partitioning between the two states considered.

**Figure 6:**
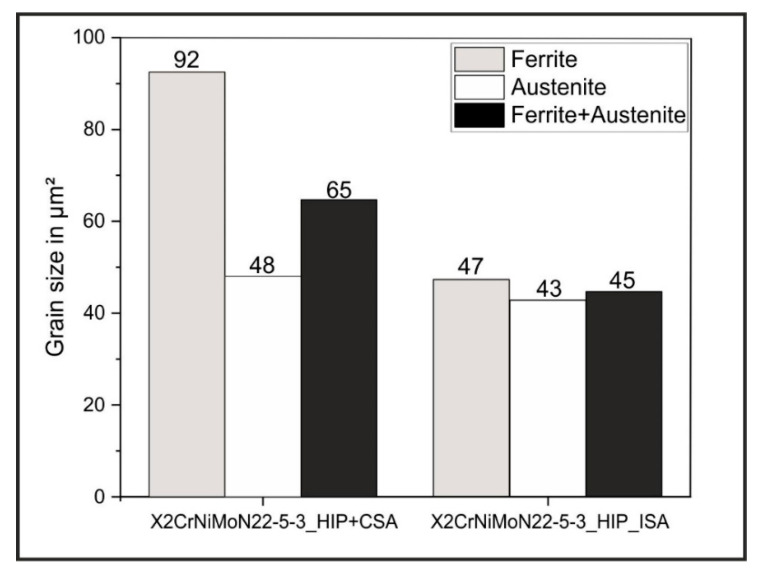
Grain sizes of the ferrite and austenite and overall grain sizes (ferrite + austenite) of the X2CrNiMoN22-5-3_HIP+CSA and X2CrNiMoN22-5-3_HIP_ISA steels.

In addition to the EBSD phase images, Figure 4b,d also shows the grain size mappings of the two material states considered. To better compare the average grain sizes, the mean ferritic and austenitic grain sizes, as well as the mean overall grain size, are shown as a function of the material condition in Figure 6. On average, the X2CrNiMoN22-5-3_HIP+CSA steel had larger austenite (48 µm^2^) and ferrite (92 µm^2^) grain sizes than the X2CrNiMoN22-5-3_HIP_ISA steel (austenite: 43 µm²; ferrite: 47 µm^2^). In addition, it was observed that the ferrite grains were larger, overall, in both conditions. The difference between the ferritic and austenitic grain sizes was clearly more pronounced in the X2CrNiMoN22-5-3_HIP+CSA steel.

The generally coarser grains of the subsequently conventionally solution-annealed steel, X2CrNiMoN22-5-3_HIP+CSA, can mainly be attributed to the additional heat input from the subsequent solution annealing. During the 60 minutes, at a temperature of 1075 °C, grain growth took place, leading to a coarsening of the microstructure compared to the X2CrNiMoN22-5-3_HIP_ISA steel that was only subjected to HIP. Since, according to the Hall–Petch relationship, a fine microstructure increases the mechanical properties of toughness and yield strength, a finer microstructure is preferable to a coarser one [25]. This results in an advantage of the integrated solution annealing within the HIP process compared to the process of “HIP + subsequent solution annealing,” as investigated by Barros et al., for example [8].

In conclusion, it can be stated that, as a result of the increased pressure at work in the HIP process, there is a slight stabilization of the austenite. Furthermore, it was shown that integrated solution annealing is possible within the HIP process. This not only saves time and energy, but also prevents grain growth, which takes place in subsequent solution annealing at an atmospheric pressure.

### 3.3. Prevention of the Sigma Phase Precipitation by HIP Quenching

In Section 3.2, by analyzing the duplex stainless steel X2CrNiMoN22-5-3, it was shown that densification and solution annealing integrated with HIP are possible. This section investigates whether the URQ function is also sufficient for suppressing the sigma phase formation of a steel that is more sensitive to sigma phase formation compared to X2CrNiMoN22-5-3. For this purpose, the steel X2CrNiMoCuWN25-7-4 was compacted by HIP and subsequently subjected to integrated heat treatment. For the detection of the sigma phase, EBSD investigations, as well as impact testing, were carried out. In addition, the temperature measured in situ inside the HIP capsule was used to provide information about whether the cooling rate was sufficient for bypassing the sigma phase formation or not. The results are shown and discussed in this section to conclude whether integrated solution annealing within the HIP process is possible. For this purpose, the sigma phase formation is first considered in general, and then the sigma phase formation in the steel under consideration is discussed on the basis of the results.

**Figure 7:**
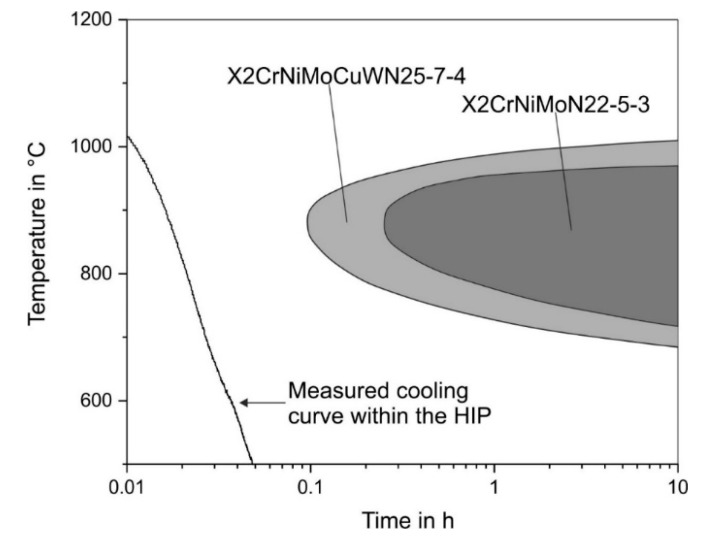
Isothermal time–temperature phase diagram for the sigma phase formation in the duplex steels X2CrNiMoN22-5-3 and X2CrNiMoCuWN25-7-4 [6]. The measured cooling curve within the HIP was added subsequently.

In general, the formation of a phase during cooling, starting from an annealing temperature, can be predicted by considering an isothermal time–temperature diagram, as shown in Figure 7**.** Here, both the time- and temperature-dependent sigma phase formations in the steels X2CrNiMoN22-5-3 and X2CrNiMoCuWN25-7-4 are shown, based on the literature. The formation of the sigma phase takes place most rapidly in a temperature range between 850 °C and 900 °C and occurs earlier in the X2CrNiMoCuWN25-7-4 steel, indicating a higher sensitivity to sigma phase formation of this steel compared to the X2CrNiMo22-5-3 steel [23,26]. This fact is why the steel X2CrNiMoCuWN25-7-4 was chosen to address the present research question regarding the sigma phase suppression during URQ. In addition to the sigma phase noses, the diagram in Figure 7 also illustrates the temperature measured inside the capsule during cooling as a function of time. With this measured cooling rate of about 4 K/s, it can be seen that quenching by URQ is sufficiently quick enough to suppress the sigma phase formation. However, the temperature was measured at the edge of the capsules, as shown in Figure 1; hence, this alone is not proof that there truly is no sigma phase in the microstructure. In order to enable strong statements about the actual absence of the sigma phase to be made, the investigations related to the microstructure and the mechanical properties are discussed below.

**Figure 8:**
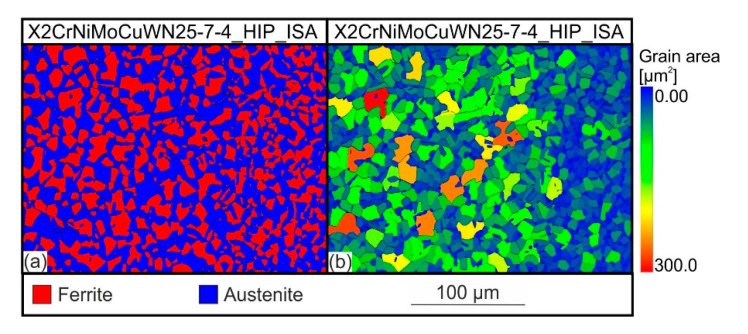
EBSD phase images **(a)** and grain size mappings **(b)** of X2CrNiMoCuWN25-7-4_HIP_ISA steel.

**Figure 9:**
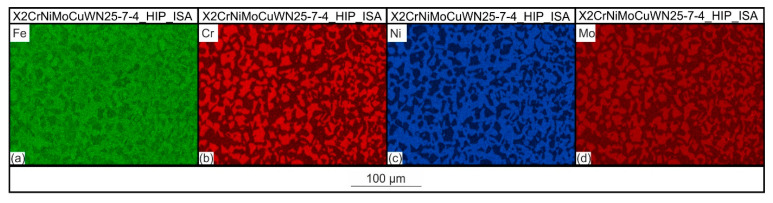
Element distributions of the X2CrNiMoCuWN25-7-4_HIP_ISA steel determined by EDS: (**a**–**d**).

Figure 8 shows the phase image and the grain size mapping resulting from the EBSD measurements of the X2CrNiMoCuWN25-7-4 steel, which was first HIP-densified, then solution-annealed within the HIP process, and subsequently quenched using URQ (integrated solution-annealed condition). The EBSD phase analysis shows a two-phase microstructure of the austenite and ferrite and did not detect the sigma phase. The austenite content resulting from the EBSD investigations was 54.5 vol.%. In some regions, a dispersive distribution of the ferrite within the austenitic matrix was detected. However, the majority of the considered area showed a duplex-typical ferritic-austenitic microstructure, in which the ferrite and austenite grains were evenly distributed. From the grain size mapping in Figure 8b, it can be seen that the grain area size of the ferrite, with a value of 32,55 µm^2^, was slightly larger than that of the austenite, with 28.95 µm^2^. The elemental distributions associated with the EBSD mapping are shown in Figure 9, which demonstrate that, similar to the X2CrNiMoN22-5-3 steel, the ferrite-stabilizing elements Cr and Mo were more abundant in the ferrite, and the austenite-stabilizing element Ni was found in a higher concentration in the austenite. The reason for the different elemental distributions between the austenite and ferrite has already been explained in Section 3.2, and it also applied to the duplex steel considered here. An important aspect that should be explained at this point, however, is the elemental distribution of the elements Cr and Mo. In principle, the sigma phase increased the Cr and Mo concentrations, which is why these distributions, in addition to the EBSD mapping, offer an important clue with which the sigma phase could be detected. Since there were no further Cr and Mo increases, apart from the concentration differences between the austenite and ferrite, the absence of the sigma phase could be confirmed on the basis of the EDS measurements.

On the basis of the EBSD and EDS examinations, the sigma phase was not detected, but the existence of the sigma phase cannot fully be excluded in the case where nanosized precipitation occurs.

**Figure 10:**
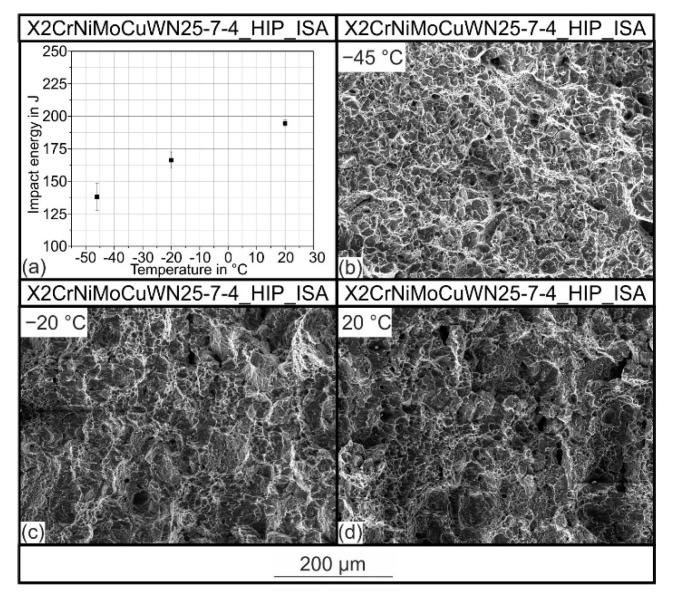
Results from the Charpy impact testing: (**a**) impact energy as a function of the test temperature; SEM image of the fracture surface at (**b**) T = −45 °C, (**c**) T = −20°C, and (**d**) T = 20 °C.

The reason why the sigma phase is undesirable in duplex stainless steels is its embrittling effect. Even a sigma phase volume content of 1 vol.% results in an enormous decrease in the steel’s impact energy and tensile strength [26]. For this reason, the sigma phase’s existence must also be verified by characterizing the mechanical properties in addition to imaging methods such as EBSD and EDS. For this reason, impact tests at different temperatures were carried out. The results are shown in Figure 10a. At a temperature of 20 °C, an impact energy of 195 J was detected. As the temperature decreased, this value dropped to 166 at −20 °C and 138 J at −45 °C. At the same time, measurement scattering was observed, which increased with decreasing temperature. In many areas of application of X2CrNiMoCuWN25-7-4 steel, the prescribed minimum value for the notched impact energy is 50 J at −46 °C [27,28]. This value was clearly exceeded in the experiments. In addition, the fracture surfaces of the materials tested at different temperatures are shown in Figure 10b–d. The fracture surface examinations indicated that a ductile honeycomb fracture was present, and no sigma phase embrittlement was detected. Finally, it can be concluded that the cooling rates achieved during quenching by URQ were sufficient for preventing the formation of the brittle sigma phase.

## 4. Conclusions

This work has shown that HIP densification with integrated solution annealing followed by rapid quenching using the URQ method is feasible for the production of duplex stainless steels. The following conclusions can be drawn from the results:

- The EBSD and XRD investigations, as well as magneto-inductive measurements using Feritscope^®^, showed that the pressure of 170 MPa applied during the HIP treatment led to the stabilization of the austenite in the X2CrNiMoN22-5-3 duplex stainless steel. In particular, the measurements obtained using the Feritscope^®^, where the largest sample volume was measured (the most representative results for the phase quantification), were in strong agreement with CALPHAD calculations.
- The grain size of the X2CrNiMoN22-5-3 duplex stainless steel solution annealed within the HIP process was smaller than that of the steel subsequently solution-annealed at an atmospheric pressure, which makes the integrated solution annealing within the HIP process attractive not only from an economic and ecologic point of view, but also with regard to the better mechanical properties of the steel due to its finer grains.
- The URQ technique used in the integrated solution annealing resulted in sufficiently high cooling rates to prevent the sigma phase formation of the sigma phase-sensitive steel X2CrNiMoCuWN25-7-4. This was proven by in situ temperature measurements taken within the HIP capsule, EBSD and EDS investigations of the resulting microstructure, and by means of the Charpy impact tests.

## Figures and Tables

**Table 1:** Chemical composition of the starting materials in mass%, measured by means of optical emission spectrometry.

Material	C	Cr	Ni	Mo	W	Cu	N	Mn	Si	V
X2CrNiMoN 22-5-3	0.02	21.8	5.4	3.1	-	0.1	0.2	1.0	0.7	-
X2CrNiMoCuWN 25-7-4	0.02	25.1	5.9	2.8	0.3	2.1	0.3	9.6	0.4	0.1

**Table 2:** Parameters used for the densification and solution annealing of the X2CrNiMoN22-5-3 and X2CrNiMoCuWN25-7-4 powder. *Quenching medium.

	Densification				Integrated SA				Post-HIP SA			
	Heating Rate in K/min	p_hold,1_ in MPa	T_hold,1_ in °C	t_hold,1_ in min	p_hold,2_ in MPa	T_hold,2_ in °C	t_hold,2_ in min	QM *	p_hold,3_ in MPa	T_hold,3_ in °C	t_hold,3_ in min	QM *
X2CrNiMoN22-5-3_HIP+CSA	40	170	1150	180	170	1075	60	URQ **	0.1	1075	60	Water
X2CrNiMoN22-5-3_HIP_ISA	40	170	1150	180	170	1075	60	URQ **	-	-	-	-
X2CrNiMoCuWN25-7-4_HIP_ISA	40	170	1150	180	170	1040	60	URQ **	-	-	-	-

* Quenching medium, ** uniform rapid quenching (100 MPa).

## Data Availability

Not applicable.
